# ZNF282 (Zinc finger protein 282), a novel E2F1 co-activator, promotes esophageal squamous cell carcinoma

**DOI:** 10.18632/oncotarget.2630

**Published:** 2014-10-24

**Authors:** So-Young Yeo, Sang Yun Ha, Eun Ji Yu, Keun-Woo Lee, Jeong Hoon Kim, Seok-Hyung Kim

**Affiliations:** ^1^ Departmentof Health Sciences and Technology, Samsung Advanced Institute for Health Sciences and Technology, Sungkyunkwan University, Seoul, Korea; ^2^ Department of Pathology, Samsung Medical Center, Sungkyunkwan University School of Medicine, Seoul, Korea; ^3^ Samsung Biomedical Research Institute, Samsung Medical Center, Seoul, Korea

**Keywords:** ZNF282, E2F1, Cell cycle, Esophageal squamous cell carcinoma, Prognosis

## Abstract

Zincfinger protein 282 (ZNF282) is a newly identified transcription factor and little is known about its expression and function. Originally, ZNF282 is known to bind U5RE (U5 repressive element) of HLTV-1 (human T cell leukemia virus type 1) with a repressive effect. Recently we reported that ZNF282 functions as an estrogen receptor co-activator and plays an essential role in breast tumorigenesis. Although these results suggest the possible role of ZNF282 in cancers, clinical significance and function of ZNF282 are completely unknown in most of cancers. Here we found that ZNF282 was frequently overexpressed in esophageal squamouscell carcinoma (ESCC) (n=165) compared with normal esophageal epithelium and its overexpression was correlated with adverse clinical outcome. Multivariate survival analysis indicated that ZNF282 expressionwas an independent prognostic predictor for poor survival in ESCC (HR: 2.56 (95% CI 1.54-4.26), *p*<0.001). In addition, depletion of ZNF282 inhibited the cell cycle progression, migration, and invasion of ESCC cells and reduced the tumorigenicity of ESCC xenograft in nude mouse. We further showed that ZNF282 is required for E2F1-mediated gene expression in ESCC cells. Thus, ZNF282 is E2F1 co-activator involved in ESCC and elevated expression of ZNF282 is an independent adverse prognostic factor in ESCC.

## INTRODUCTION

Esophageal squamous cell carcinoma (ESCC) is common among Asian populations and one of the most aggressive malignant tumors, with a 5-year survival rate of only about 10% [1-3]. Given the poor prognosis of ESCC and its high incidence rate [4, 5]. It is increasingly important to understand the initiation and progression of this type of cancer and to identify the associated prognostic factors.

Zinc finger protein 282, (ZNF282, also known as HUB1), was originally identified as a HTLV-I (human T-cell leukemia virus type I) U5RE (U5 repressive element) binding protein [6]. Recently, we have shown that ZNF282 interacts with estrogen receptor α (ERα) and functions as an ERα co-activator in breast cancer cells [7]. However, little is known about its expression and roles in other human cancers.

Recent studies have highlighted the link betweenmisregulation of transcriptional co-activators and carcinogenesis of various cancers [8]. To date, several transcriptional co-activators have been reported to befrequently overexpressed in ESCC including deleted in breast cancer 1 (DBC1), p300, β-catenin, amplified in breast cancer 1 (AIB1) [9-13]. Thus, we postulated that ZNF282, one of transcriptional coactivator, may also play an important role in tumor progression of ESCC.

To prove this hypothesis, we evaluated the expression of ZNF282 in 165 ESCC and its association with clinicopathologic parameters and its prognostic significance. We also assessed the effect of ZNF282 depletion and overexpression on the function of cancer cells through *in vitro* and *in vivo*experiments. Altered expression of cell cycle regulatory genes has beenreported to be associated with the development and prognosis of ESCC [14]. Multiple components, such as cyclins, cyclin-dependent kinases (Cdks), Cdk inhibitors, retinoblastoma (Rb) and E2F family transcription factors, are involved in the transition of cell cycle phases [14-16]. Here, we provide several lines of evidence that ZNF282 can function as acoactivator for one of the key cell cycle-regulating transcription factors, E2F1. Therefore, our results suggest a novel mode of ZNF282 function in ESCC and directly link ZNF282 to cell cycle control mechanisms.

## RESULTS

### ZNF282 protein expression in normal esophagus and esophageal squamous cell carcinoma

**Figure 1:**
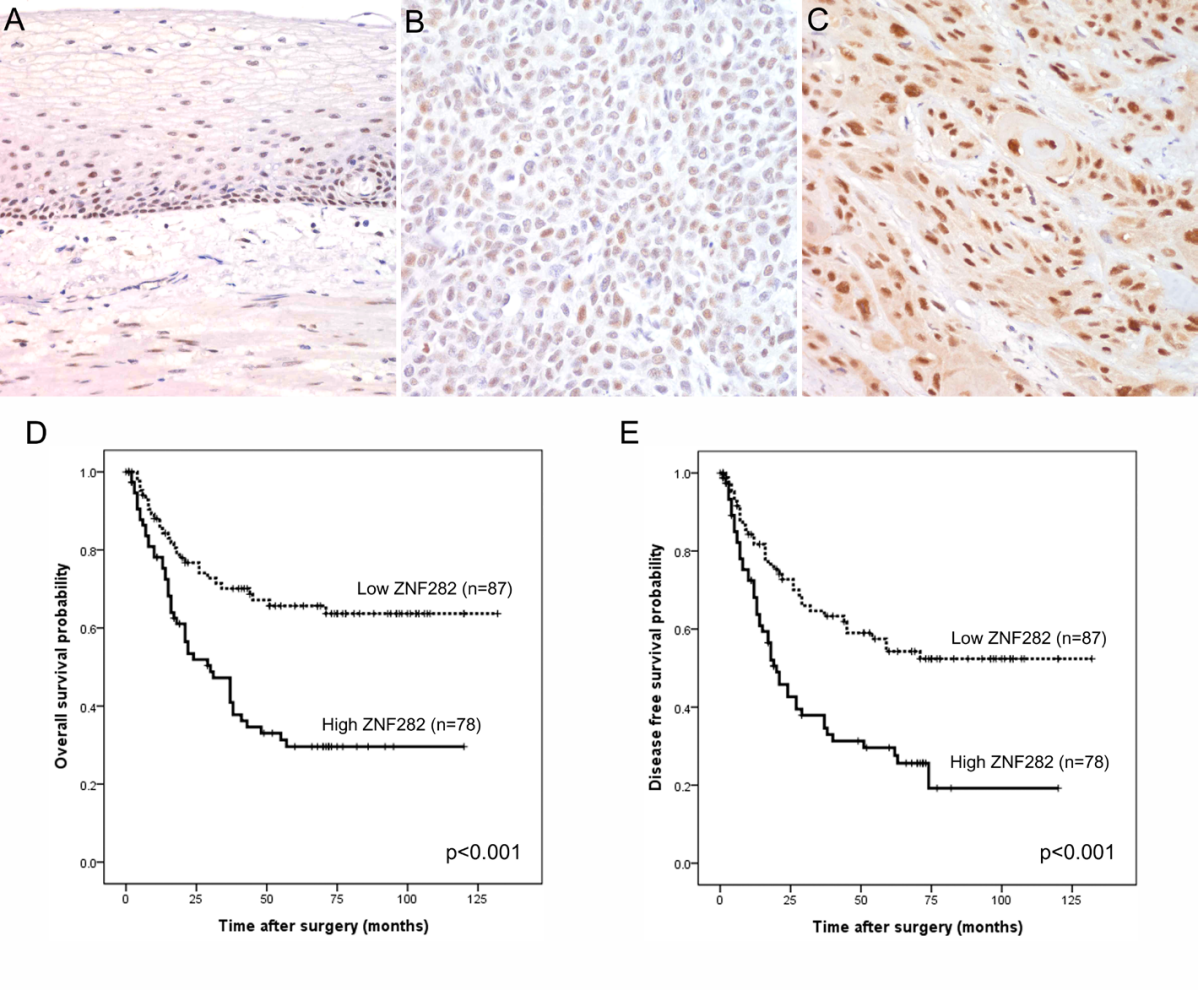
**(A-C) Representative immunohistochemical staining in normal esophageal squamous epithelium and esophageal squamous cell carcinoma for ZNF282:** (A) weak expression in normal squamous epithelium (B) low expression in esophageal squamous cell carcinoma (C) high expression in esophageal squamous cell carcinoma. (D-E) Kaplan-Meier curves illustrating overall survival (D) and relapse-free survival (E) among esophageal squamous cell carcinoma patients based on ZNF282 expression status. Both overall and disease-free survival are significantly worse in patients with a high expression of ZNF282 (*P*<0.001).

ZNF282 staining was mostly observed in the nucleus (Figure. 1A-C)and we semi-quantitatively evaluated nuclear expression of ZNF282 in ESCC and normal esophagus. The overexpression of ZNF282 was more frequent in ESCC than in normal epithelium with a statistical significance (47.2% vs 5.7%, *p*<0.001, Supplementary Table 1). The associations between ZNF282 expression and clinicopathologic variables are shown in Table 1. Of note, the high ZNF282 expression was significantly correlated with advanced T stage (p=0.019). This correlation between high ZNF282 expression and advanced T stage was more distinctly observed in the small size (<4cm) tumor group (p=0.0005).

**Table 1:** Relations between ZNF282 expression and clinicopathologic features in esophageal squamous cell carcinoma

		ZNF282 expression		
	Total	Low	High	
	n=165	n=87 (52.7%)	n=78 (47.3%)	
Age (years)				
<65	38 (23.0)	19 (21.8)	19 (24.4)	0.701
≥65	127 (77.0)	68 (78.2)	59 (75.6)	
Gender				
Female	6 (3.6)	4 (4.6)	2 (2.6)	0.685^†^
Male	159 (96.4)	83 (95.4)	76 (97.4)	
Tumor size (cm)				
<4	70 (42.4)	39 (44.8)	31 (39.7)	0.509
≥4	95 (57.6)	48 (55.2)	47 (60.3)	
Differentiation				
W/D	27 (16.4)	16 (18.4)	11 (14.1)	0.457
M/D	108 (65.5)	58 (66.7)	50 (64.1)	
P/D	30 (18.2)	13 (14.9)	17 (21.8)	
T stage				
T1	33 (20.0)	23 (26.4)	10 (12.8)	0.019^‡^
T2	29 (17.6)	17 (19.5)	12 (15.4)	
T3	93 (56.4)	42 (48.3)	51 (65.4)	
T4	10 (6.1)	5 (5.7)	5 (6.4)	
N stage*				
N0	59 (37.6)	31 (37.3)	28 (37.8)	0.496^‡^
N1	41 (26.1)	24 (28.9)	17 (23.0)	
N2	31 (19.7)	17 (20.5)	14 (18.9)	
N3	26 (16.6)	11 (13.3)	15 (20.3)	
M stage				
M0	144 (87.3)	77 (88.5)	67 (85.9)	0.616
M1	21 (12.7)	10 (11.5)	11 (14.1)	
Chemotherapy				
Absent	54 (32.7)	28 (32.2)	26 (33.3)	0.875
Present	111 (67.3)	59 (67.8)	52 (66.7)	
Radiation therapy				
Absent	95 (57.6)	50 (57.5)	45 (57.7)	0.977
Present	70 (42.4)	37 (42.5)	33 (42.3)	
Recurrence				
Absent	78 (47.3)	51 (58.6)	27 (34.6)	0.002
Present	87 (52.7)	36 (41.4)	51 (65.4)	
Death				
Absent	89 (53.9)	59 (67.8)	30 (38.5)	<0.001
Present	76 (46.1)	28 (32.2)	48 (61.5)	

* 8 cases of unsastisfactory for minimal number of evaluated lymph nodes, were excluded in analysis.

† Fisher exact test

‡ Cochran Armitage test, otherwise Pearson Chi-square test.

### Overexpression of ZNF282 is associated with shorter survival time in ESCC patients

Both OS (Figure. 1D) and DFS rate (Figure. 1E) of patients with high expression of ZNF282 were significantly lower than those of patients with low expression (*p*<0.001 for both). Moreover, high expression of ZNF282 was identified as an independent prognostic factor for both OS [HR: 2.>56 (95% CI 1.54-4.26), p<0.001] and DFS [HR:2.28 (95% CI 1.43-3.62, p<0.001], in the multivariate analysis (Table 2).

**Table 2:** Multivariate Cox proportional hazard model analyses by ZNF282 expression

Characteristic	Category	Overall survival			Disease-free survival		
HR	95% CI	p-value	HR	95% CI	p-value
Age	≥65 vs <65	1.25	0.67-2.35	0.487	1.18	0.67-2.09	0.568
Tumor size	≥4cm vs <4cm	1.11	0.61-2.01	0.736	0.97	0.56-1.67	0.899
Differentiation	M/D vs W/D	1.36	0.57-3.21	0.489	1.23	0.56-2.69	0.611
	P/D vs W/D	2.21	0.92-5.29	0.075	1.57	0.67-3.67	0.301
T stage	2 vs 1	1.11	0.37-3.35	0.855	0.95	0.36-2.52	0.917
	3 vs 1	1.60	0.62-4.11	0.333	1.27	0.55-2.89	0.575
	4 vs 1	7.00	2.01-24.15	<0.001	3.33	1.00-11.09	0.051
N stage	2 vs 1	1.45	0.66-3.17	0.36	1.64	0.79-3.40	0.187
	3 vs 1	2.82	1.27-6.24	0.011	2.90	1.37-6.10	0.005
	4 vs 1	6.34	2.93-13.75	<0.001	5.88	2.77-12.50	<0.001
M stage	1 vs 0	0.92	0.45-1.91	0.827	1.19	0.63-2.25	0.584
Chemotherapy	Positive vs Negative	0.78	0.45-1.35	0.38	0.95	0.57-1.59	0.843
Radiation therapy	Positive vs Negative	1.56	0.93-2.60	0.091	1.85	1.14-2.99	0.013
ZNF282 expression	High vs Low	2.56	1.54-4.26	<0.001	2.28	1.43-3.62	0.001

### ZNF282 expression in ESCC cell lines

**Figure 2:**
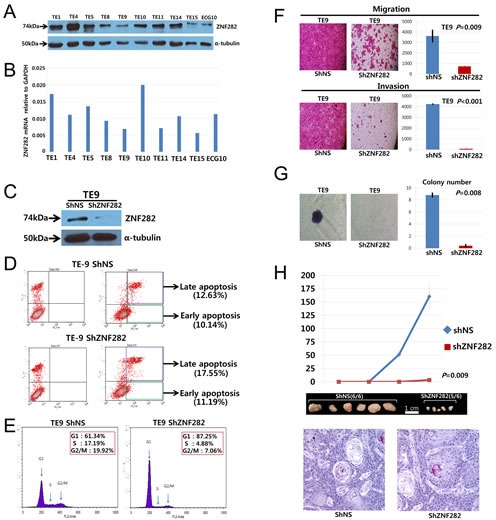
(A-B)ZNF282 protein expression and mRNA expression in ESCC cell lines. (A) Results of western blotting analysis. (B) Amplification curve for the PCR product by real time RT-PCR. (C) Depletion of ZNF282 in TE9 by transfection of siRNA against ZNF282. (D-E) Knockdown of ZNF282 expression induces late apoptosis (D) and cell cycle arrest in ESCC cellline (E). (F-G) The effect of ZNF282 depletion on the proliferation, migration, and invasion of esophageal squamous cell carcinoma cell line (TE9). (F) Migration and invasion of TE9 are greatly reduced after siRNAtransfection. Scrambled siRNA was used as a negative control. (G) Colony formation assay shows that tumorigenic ability is also significantly reduced in cancer cells when siZNF282 is transfected compared to when siNS was transfected. (H) Knockdown of ZNF282 inhibitedgrowth of ESCC *in vivo.* Knockdown of ZNF282 significantly inhibits growth of TE9 ESCC cells (mean tumor volume: 3.33 mm^3^) compared to the control group (mean tumor volume: 160.42 mm^3^)(p=0.009). (I) Histologic examination confirmed that the TE9 xenograft tumor is a well-differentiated squamous cell carcinoma.

Western blotting revealed single and strong protein bands at approximately the 74kDa (Figure. 2A)and indicated moderate to strong expression of ZNF282 protein in all these cell lines. qRT-PCR showed that ZNF282 mRNA was also highly upregulated in all these ESCC cell lines (Figure. 2B), and this result is in line with that of western blotting. These resultsin ESCC cell lines are very well consistent with frequent overexpression of ZNF282 in ESCC patients' tissues.

### Knockdown of ZNF282 expression in ESCC cell line induced late apoptosis and cell cycle arrest

In order to determine the role of ZNF282 during tumor progression of ESCC, we designed shRNA against ZNF282 and confirmed that ZNF282 expression was greatly reduced by shRNA treatment (Figure. 2C). Because ZNF282 overexpression is rare in normal esophageal epithelium and far more frequent in ESCC (Supplementary Table 1), it is suspected that ZNF282 may influence on the cell survival and cell cycle. ZNF282 knockdown in TE9 cells increased late apoptotic compartment (17.55 %) compared to control group (12.63 %), while no significant difference in the proportion of early apoptotic cells between shZNF282 group (11.19 %)and control group (10.14 %) (Figure. 2D). In addition, the proportion of cells in G1 phase was significantly increased in shZNF282 treated TE9 cells (87.25%) compared to control shNS treated TE9 cells (61.34%) whereas the proportions of cells in S phase and G2/M phase were markedly decreased in shZNF282 treated group (4.88% in S phase and 7.06% in G2/M phase) compared to control group (17.19% in S phase and 19.92% in G2/M phase) (Figure. 2E). These results suggest that ZNF282 suppresses apoptosis and cell cycle arrest of ESCC.

### Knockdown of ZNF282 expression reduced migration, invasion and tumorigenesis of ESCC *in vitro*

Because ZNF282 overexpression in ESCC is correlated with tumor invasiveness (advanced T stage), it is suspected that ZNF282 may enhance the invasive ability of tumor. Therefore, we assessed the effect of ZNF282 on migration/invasion of ESCC. Both migration and invasion of TE9 cells were significantly reduced in shZNF282-treated group compared to control group (Figure. 2F). *In vitro*anchorage independent tumorigenic activity of TE9 ESCC cells was also significantly decreased in shZNF282-treated group as shown in soft agar colony formation assay (Figure. 2G). These results suggest that ZNF282 promotes migration / invasion and facilitates anchorage independent tumor growth of ESCC. This is well consistent with our clinical observation previously mentioned that ZNF282 overexpression was positively correlated with advanced T stage inESCC because T stage indicates the extent and depth of direct tumor invasion (Table 2).

### Knockdown of ZNF282 expression inhibited growth of ESCC *in vivo*

We next investigated whether ZNF282 promotes tumor growth in *in vivo* model as well as *in vitro*experiment. To this end, TE9 ESCC cells transduced with lentivirus expressing either shZNF282 or shNS (control) were subcutaneously injected into six immune-compromised nude mice, respectively. Tumor growths in these mice were followed up for eight weeks and mice were sacrificed to measure the tumor growth. Knockdown of ZNF282 significantly inhibited growth of TE9 ESCC cells (mean tumor volume : 3.33 mm^3^) compared to control group (mean tumor volume : 160.42 mm^3^) (p=0.009) (Figure. 2H). Histologic examination confirmed that TE9 xenograft tumor was well differentiated squamous cell carcinoma (Figure. 2I).

### Rescue overexpression of ZNF282 in ZNF282 depleted ESCC cell line induced increase in migration, invasion, colony formation and sphere formation

We designed lentivirus-expressing human FLAG-ZNF282 and FLAG control vector and transduced them to TE9 ESCC cells which had been previously treated with shZNF282 (Supplementary Figure 1A). Rescue ZNF282 overexpression in ZNF282-depleted cells induced significant increases in migration, invasion, anchorage independent growth and sphere formation in ZNF282- depleted cell lines (Supplementary Figure 1B-C). These results again confirmed the functional involvement of ZNF282 in ESCC progression. In contrast, additional ZNF282 overexpression in ESCC cells that already expressed ZNF282 endogenously in high level did not induce any significant change in migration, invasion, *in vitro* tumorigeneicity (Supplementary Figure 1B-C).

### ZNF282 functions as an E2F1 co-activator

**Figure 3:**
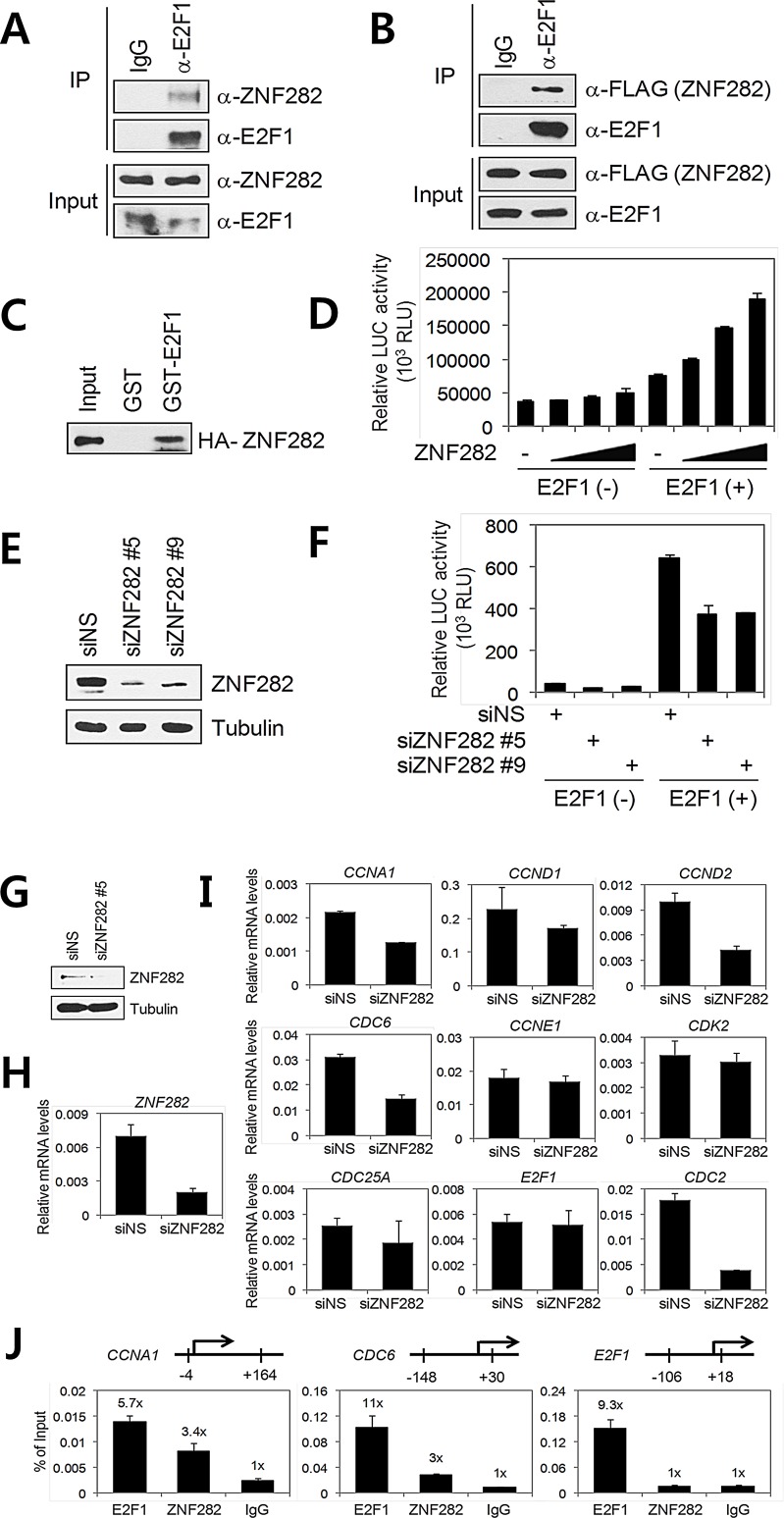
ZNF282 interacts with E2F1 and functions as an E2F1 co-activator (A)Endogenous interaction between ZNF282 and E2F1. TE10 cell lysates were immunoprecipitated with anti-E2F1 antibody or control normal IgG, followed by immunoblot with the indicated antibodies. (B) 293T cells were transfected with pSG5.HA-E2F1 and pFLAG.CMV4-ZNF282. Cell lysates were immunoprecipitated with anti-E2F1 antibody or control normal IgG. Immunoprecipitated E2F1 and coimmunoprecipitated ZNF282 were detected byanti-FLAG antibody and anti-E2F1 antibody, respectively. (C) *In vitro*translated HA-tagged ZNF282 was incubated with recombinant GST-E2F1. Bound proteins were analyzed by immunoblot with anti-HA antibody. (D) Coactivator activity of ZNF282 for E2F1. 293T cells were transfected with pSG5.HA-E2F1 (20 ng) and 5xE2F-TA-LUC reporter (200 ng) in combination with various amounts (200, 400, 600 ng) of pSG5.HA- ZNF282. Cell extracts were assayed for luciferase activity. Results shown are mean and SD of triplicate points. (E and F) ZNF282 is required for the transcriptional activity of E2F1. 293T cells were transfected with pSG5.HA-E2F1, 5xE2F-TA-LUC, and 40 pmol of ZNF282 siRNA#5, #9 or NS (non-specific) siRNA duplex. 72 h after transfection, cells extracts were harvested for immunoblot analysis (E) and luciferase assays (F). (G-I) Requirement of ZNF282 for the expression of a subset of E2F1 target genes and recruitment of ZNF282 to selective E2F1 target genes. (G, H, and I) TE10 cells were transfected with 40 pmol of ZNF282 siRNA#5or NS siRNA duplex. 72 h after transfection, protein extracts and totalRNA were prepared. Protein levels were monitored by immunoblot using the indicated antibodies (G). Total RNA was examined by real-time quantitative RT–PCR (qRT–PCR) analysis with primers specific for the indicated mRNAs (H and I). Results shown were normalized to b-actin mRNAlevels and are means ± standard deviation (n=3). (J) ChIP assay. Crosslinked, sheared chromatin from TE10 cells was immunoprecipitated with the indicated antibodies. Quantitative PCR (qPCR) analyses were performed using primers specific for the indicated promoters. The results are shown as percentage of input and are means ± standard deviation (n=3).

E2F1 is a transcription factor which plays a critical role in cell cycle progression, cell proliferation, apoptosis, and metastasis of cancer cells [17]. Because ZNF282 functions as a transcription co-activator [7]and given that ZNF282 is required for cell cycle progression and survival of ESCC cells, we next investigated possible physical and functional interactions of ZNF282 with cell cycle-regulating transcription factor E2F1. We first examined the association between endogenous ZNF282 and E2F1 in TE10 cells by coimmunoprecipitation (CoIP)assays. As shown in Figure 3A, ZNF282 bound to E2F1. Similarly, ZNF282 was coimmunoprecipitated specifically with E2F1 from extracts of transiently transfected 293T cells (Figure 3B). In addition, *in vitro* GST pull-down assays confirmed the interaction between ZNF282 and E2F1 (Figure 3C), suggesting that ZNF282 interacts directly with E2F1.

**Figure 4:**
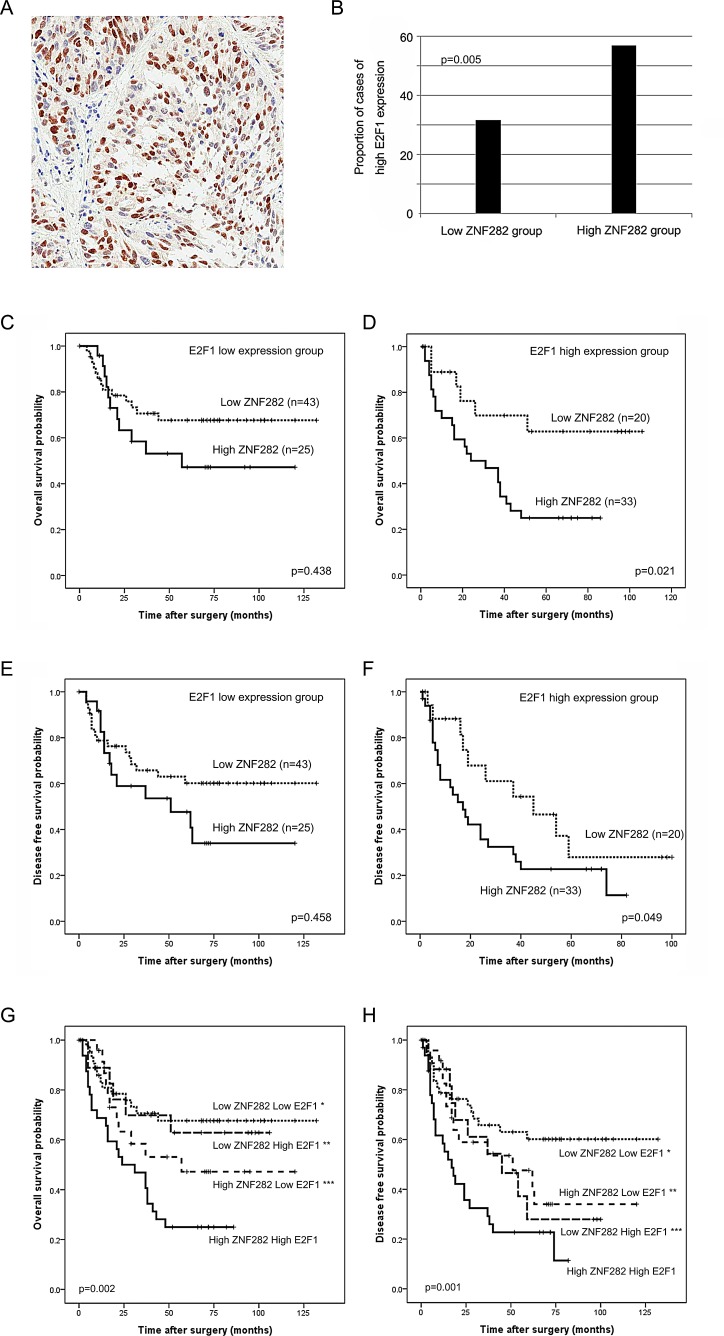
(A) E2F1 is stained in the nucleus of esophageal squamous cell carcinoma cells. (B)E2F1 expression is more frequently found in ZNF282 high-expression group than in low-expression group (62.3% vs 36.8%, p=0.005). (C-F) Kaplan-Meier curves illustrating overall survival (C-D) and disease freesurvival (E-F) among esophageal squamous cell carcinoma patients based on ZNF282 expression status in E2F1 high or low expression groups. Patients with ZNF282 high expression shows significantly shorter survival rate in only E2F1 high expression group. (G-H) Kaplan-Meier curves illustrating overall survival (G) and disease free survival (H) among esophageal squamous cell carcinoma patient groups based on combination of expression pattern of ZNF282 and E2F1. ZNF282-high E2F1-high group shows shortest overall survival (^*^ vs low ZNF282 low E2F1 group, p=0.001; ^**^ vs low ZNF282 high E2F1, p=0.051; ^***^ vs high ZNF282 low E2F1, p=0.021) and disease free survival (^*^ vs low ZNF282 low E2F1 group, p=0.001; ^**^ vs low ZNF282 high E2F1, p=0.027; ^***^ vs high ZNF282 low E2F1, p=0.049).

In transient transfection assays, overexpressionof ZNF282 enhanced expression of a reporter plasmid controlled by E2F1 binding sites in a dose-dependent and E2F1-dependent manner in 293T cells (Figure 3D). To assess the functional involvement of endogenous ZNF282 in E2F1-mediated transcription, the expression of ZNF282 was reduced by RNAinterference (RNAi). To avoid possible off-target effects caused by theZNF282 RNAi, we used two different siRNA sequences that target different regions of ZNF282 mRNA (Figure 3E). As shown in Figure 4F, depletion of ZNF282 reduced the transcriptional activity of E2F1, suggesting that ZNF282 functions as an E2F1 co-activator and is requiredfor full transcriptional activity of E2F1.

### ZNF282 is required for the expression of a subset of E2F1 target genes and is recruited to selective E2F1 target genes

To further assess the physiological role of ZNF282 as an E2F1 co-activator, we determined the effect of ZNF282 depletion on the expression of the endogenous E2F1 target genes including E2F1 itself. When ZNF282 protein and mRNA levels were specifically reduced in TE10 cells by siRNA transfection (Figure 3G and 3H), the expression of well characterized E2F1 target genes such as CCND2, CCNA1, CDC2, and CDC6 was significantly inhibited compared with the results using a NS siRNA (Figure 3I). Interestingly, ZNF282 depletion showed little or no effect on the expression of CCND1, CCNE1, CDK2, CDC25A, and E2F1, suggesting that ZNF282 is required for the expression of a subset of E2F1 target genes.

Having demonstrated that ZNF282 depletion reduces the expression of a subset of E2F1-responsive genes, we next performed chromatin immunoprecipitation (ChIP) assays in TE10 cells to examine whether ZNF282 is recruited to the target gene promoters. As expected, E2F1 was recruited to the E2F1 binding sites associated with the CCNA1, CDC6, and E2F1 gene promoters (Figure 3J). ZNF282 was also recruited to the CCNA1 and CDC6 gene promoters, indicating that ZNF282 is directly involved in the transcriptional regulation of endogenous E2F1 target genes. However, no recruitment of ZNF282 was detected on E2F1 binding sites associated with the E2F1 gene. These results are consistent with the data that ZNF282 did not significantly affect E2F1 gene expression (Figure 3I) and strongly suggest that ZNF282 is directly involved in the expression of a specific subset of E2F1 target genes.

### The prognostic effect of ZNF282 was more significant in E2F1 high-expression group than in low-expression group

Because ZNF282 functions as a transcription co-activator of E2F1, which is a critical player in tumor progression, we investigated whether the prognostic effect of ZNF282 in ESCC was influenced by E2F1. E2F1 expression was frequently observed in the nucleus of ESCC cells (56/126, 44.4%) and overlapped with ZNF282 expression in 27.3% of ESCC cases. E2F1 overexpression was more frequently found in ZNF282 high-expression group than in low-expression group (56.9% vs 31.7%, p=0.005) (Figure 4A-B). The prognostic effect of ZNF282 in OS and DFS was more significant in E2F1 high-expression group than in low-expression group (OS, p=0.021 vs p=0.438; DFS, p=0.049 vs p=0.458) (Figure 4C-F). Furthermore, the ESCC patients with high expression of both ZNF282 and E2F1 (33/121, 27.3%) showed the worst overall and disease-free survival with statistical significance (Figure 4G-H). On the other hand, the ESCC patients with low expression of both ZNF282and E2F1 (43/121, 35.5%) exhibited the best prognosis of all subgroup (Figure 4G-H). These results demonstrated that the prognostic effect of ZNF282 was dependent on E2F1, which is strongly consistent with the previous data showing the role of ZNF282 as a noble co-activator of E2F1.

## DISCUSSION

In present study, we found that ZNF282 was frequently overexpressed in ESCC compared with normal esophageal epithelium (47.2% vs 5.7%, p<0.001) and its overexpression was an unfavorable prognostic indicator in ESCC in both univariate and multivariate survival analysis (both p<0.001). Besides, we demonstrated that knockdown of ZNF282 induced apoptosis and cell cycle arrest as well as decreased migration, invasion, and tumorigenenicity *in vitro,*and inhibited growth of ESCC xenograft tumor in nude mice. Thus, our results indicate that ZNF282 plays an important role in tumor progression of ESCC.

The role of ZNF282 in human cancer has been very rarely studied except for our previous report [7]. In that report, we firstly presented essential roles of ZNF282 in migration, proliferation, and tumorigenicity of breast cancer cells. Despite the difference in the cancer type and organ, the present resultsare well consistent with our previous report [7]. In addition to *in vitro* and *in vivo* results, we showed the clinical relevance of ZNF282 expression in ESCC using a large cohort tissue microarray for the first time as far as we know.

To investigate the expression of ZNF282 in othertypes of cancer, we searched the public cancer microarray database ONCOMINE (http://www.oncomine.org) and found that many human malignant tumors show increased expression of ZNF282 compared with normal counterpart cells (Supplementary Figure 2). In those studies, expressionof ZNF282 mRNA was higher in esophageal, tongue, colon, liver, pancreas, lung, urinary bladder, breast, brain cancer and melanoma samples than in normal samples, suggesting that ZNF282 might play an important role in growth and tumorigenesis of a variety of cancer cells. In particular, expression of ZNF282 mRNA was significantly increased inESCC (two independent studies) and tongue squamous cell carcinoma and these results are very highly consistent with our result that ZNF282 expression is upregulated in ESCC (Supplementary Figure 2) [18].

E2F1 is the best characterized member of the E2Ftranscription family and has been identified as a key downstream targetin the pRb pathway [17, 19, 20]. E2F1 is well known for its paradoxical function as oncogene and tumor suppressor in different cancers [17]. Nevertheless, its overexpression in multiple cancers including breast cancer and ESCC has been strongly linked to cancer progression. E2F1 DNAbinding sites have been identified in the promoter regions of many genes involved in DNA replication or cell cycle control, and overexpression of E2F1 can drive cells from G1 into the S phase [21, 22]. Thus, transcriptional activation by E2F1 is important for cell cycle progression and cell proliferation. Previous studies reported that transcription factor E2F1 is expressed strongly in 59.8% of ESCC and that the overexpression is correlated with tumor progression, lymph nodemetastasis, and poor prognosis after surgery [23-25]. Another study reported that increased cell proliferation and decreased apoptosis resulting from E2F1 overexpression are associated with adverseprognosis in patients with ESCC [15]. Although there is increasing evidence that E2F1 is associated with ESCCaggressiveness and poor clinical outcomes, the underlying mechanisms ofE2F1-mediated transcriptional regulation in ESCC are not well defined.

In this study, we provided several lines of evidence that ZNF282 functions as an E2F1 co-activator in ESCC cells: ZNF282 interacted with and enhanced the transcriptional activity of E2F1; depletion of ZNF282 caused reduction in the expression of endogenous E2F1 target genes including CCND2, CCNA1, CDC2, and CDC6; ZNF282 was recruited to the promoters of CCNA1 and CDC6 genes, but not to the promoter of E2F1 gene, indicating a direct involvement of ZNF282 in transcriptional control of a subset of E2F1 target genes. ZNF282 depletion increased apoptosis and inhibited cell cycle progression at G1/S. Because E2F1 facilitates cell cycle progression by stimulating G1/S transition, these results suggest that ZNF282 has a critical role as an E2F1 co-activator in cell cycle control of ESCC cells. Interestingly, our finding suggests that ZNF282 regulates the expressionof only a specific subset of E2F1 target genes. Although the precise mechanism underlying gene-specific involvement of ZNF282 is not fully understood, one intriguing possibility is that chromatin structure surrounding E2F1 binding sites and pioneer factors for E2F1 determine ZNF282 recruitment to E2F1-regulated promoters. Recently, ANCCA has beenreported to act as a pioneer factor for E2F1 by anchoring itself at specific chromatin locations and then loading E2F1 and its coactivators [26]. Thus, it is possible that pioneer factors for E2F1 and specific chromatin structure may contribute to selective recruitment of co-activators to E2F1 binding sites. Further study will be needed to define the exact mechanism of gene-specific requirement for ZNF282 in E2F1-mediated transcription.

In conclusion, the present study suggests a rolefor ZNF282 in the prognosis of ESCC patients. To our knowledge, this isthe first report to evaluate ZNF282 expression and its effect on clinical outcome in human cancer samples. Our findings emphasize the importance of ZNF282 as a potential marker of tumor progression and its possible use in diagnosis, prognosis or development of therapeutic toolsagainst ESCC.

## MATERIALS AND METHODS

### Patients, tissue samples and construction of tissue microarray

We investigated 199 cases of normal esophagus and esophageal squamous cell carcinoma. The data were procured from surgical pathology files kept at the Pathology Department of Samsung Medical Center, Seoul, Korea: 165 cases of ESCC and 34 cases of normal esophageal tissues. The pathologic feature of specimens was classified based on the 7^th^ edition of the TNM classification (UICC). The same chemotherapy regimen was applied and allchemotherapy was conducted after surgery. Patients underwent neoadjuvant chemotherapy or radiotherapy were not included in this study. All archival materials were routinely fixed in 10% neutral-buffered formalin and embedded in paraffin. We constructed tissue microarray as described previously [27]. This retrospective study was approved by institutional review board of Samsung Medical Center and conducted in accordance with the 1996 Declaration of Helsinki.

### Immunohistochemical staining procedure

Immunostaining for ZNF282 protein and E2F1 was performed using rabbit polyclonal antibody recognizing ZNF282 specifically (HPA024374, Sigma, St. Louis, MO, USA) and E2F1 antibody (sc-251, Santa cruz, Dallas, Texas, USA). Tissue sections embedded in the microslides were deparaffinized with xylene, hydrated in serial dilutions of alcohol, and immersed in peroxidase-blocking solution (DakoREAL™ Peroxidase-Blocking Solution, DAKO) to quench endogenous peroxidase activity. The sections were then microwaved in 10mM Tris buffer (pH 9.2) for 25 minutes. The sections were then incubated with the primary antibody (dilution ratio, 1:1000 for ZNF282, 1:300 for E2F1)for 1 hour and rinsed three times successively with washing buffer. Further incubation was performed using the DAKOREAL™ EnVision™/HRP, Rabbit/Mouse (Envision) detection reagent for 1 hour at room temperature.

### Evaluation of results of the immunohistochemical staining

We used the scoring method of Sinicrope et al. [28]in the analysis of immunohistochemical stainings. The staining intensity was further classified as follows: 1 (weak), 2 (moderate), and3 (strong). The positive cells were quantified as a percentage of the total number of epithelial cells and were assigned to one of the following five categories: 0 (<5%), 1 (5%–25%), 2 (26%–50%), 3 (51%–75%), and 4 (>75%). The percentage of positivity of the tumor cells and the staining intensities were then multiplied to generate an immunohistochemistry (IHC) score for each tumor specimen. An IHC score between 0 and 6 is considered to indicate “low expression” and an IHC score between 7 and 12 is considered to indicate “high expression” for ZNF282, and an IHC score between 0 and 1 is considered to indicate “low expression” and an IHC score between 2 and 12 is considered to indicate “high expression” for E2F1. Each lesion was separately examined and scored by two pathologists (S.H.K and S.Y.H). The pathologists discussedany cases showing a discrepancy in scores until a consensus was reached.

### Cell culture and transient transfection

Human esophageal squamous carcinoma TE1, TE4, TE5, TE8, TE9, TE10, TE11, TE14, TE15, and ECG10 cell lines were purchased from RIKEN (Saitama, JAPAN) and were maintained in RPMI mediumsupplemented with 10% fetal bovine serum (FBS). 293T cells were purchased from the Korean Cell Line Bank (Seoul, Korea) and cultured in DMEM with 10% fetal bovine serum (FBS). Transient transfections and reporter gene assays were performed as described previously [7]. The empty vector was used as control for transfections of all coactivator plasmids, and equal total DNA amounts were used in all samples in a given experiment. Each experiment was repeated independently at least three times. The results shown are the means and standard deviation of triplicate points.

### Lentivirus transduction

Human embryonic kidney 293T cell lines were cultured in DMEM (Hyclone) with 10% FBS. The packaging of vector was obtained by transfection of 293T. TE9 cells were infected with a lentivirus encoding a non-specific (NS) shRNA or ZNF282 shRNA using Polybrene (Millipore) and selected with 2 ug/ml puromycin as decribed previously [7, 29]. TE9 cells were transduced with lentivirus-expressing human FLAG- ZNF282and FLAG control vector. The cells were infected with lentivirus and selected by hygromycin (Sigma-Aldrich).

### RNA interference and Real Time qRT-PCR

The depletion of ZNF282 was performed by transfection of TE9 and TE10 cells with either of two siRNA duplexes: siZNF282#5 [7]; siZNF282#9 (Dharmacon, J-011473-09). Non-specific (NS) siRNA was described previously [7]. Quantitative real-time reverse transcriptase-PCR (qRT-PCR) was performed with Brilliant SYBR Green QRT-PCR Master Mix 1-Step (Stratagene) with the ABI HT-7900 system (Applied Biosystems). Relative expression levels of the target genes were determined using the comparative Ct (ΔΔCt) method and normalized to the expression level of β-actin or GAPDH mRNA. Primers used are list as follow: ZNF282[7]; β-actin [29]; GAPDH [27]; CCND1 [30];CCND2, 5′-GAG AAG CTG TCT CTG ATC CGC A-3′ (forward) and 5′-CTT CCA GTTGCG ATC ATC GAC G-3′ (reverse); CCNA1, 5′-GGC ACA GAT GTG ATA AAT GTG ACT-3′ (forward) and 5′-CAG ATA CAG GGT CTC TGC TCG AAG-3′ (reverse); CCNE1, 5′-CAG GAT CCA GAT GAA GAA ATG GCC-3′ (forward) and 5′-GGA TGG TGC AAT AAT CCG AGG CTT-3′ (reverse); CDK2, 5′-CCC TTT CTT CCA GGA TGT GA-3′ (forward) and 5′-TCA CCC CTG TAT TCC CAG AG-3′ (reverse); CDC2, 5′-CTA GAA AGT GAA GAG GAA GGG-3′ (forward) and 5′-CAT GTA CTG ACC AGG AGG GAT-3′ (reverse); CDC6, 5′-GAT GCC AAA CTA GAA CCA ACA-3′ (forward) and 5′-CAA TCT TCG TCC CTT AAG TGT-3′ (reverse); CDC25A, 5′-GTA AGA CCT GTA TCT CGT GGC TGC-3′ (forward) and 5′-CTC TCC ATC GAG AAG GTC CAC GAA-3′ (reverse).

### Migration and invasion assay

*In vitro* Matrigelinvasion assays were done using 6.5-mm Costar transwell chambers (8-Am pore size; Corning, NY, USA). The Transwell filters were coated with appropriate Matrigel (1mg/ml) (Becton Dickinson, Franklin Lakes, NJ, USA). After the Matrigel solidified at 37°C, 1 × 10^5^ cellswere seeded onto the Matrigel. After incubation for 22 hours, the filter was gently removed from the chamber and the noninvasive cells on the upper surface were removed by wiping with a cotton swab. The cells that invaded the Matrigel and attached to the lower surface of the filter were fixed with methanol and stained with H&E solution. The number of cells attached to the lower surface of the polycarbonate filter was counted at X 400 magnification under a light microscope. The migration assay was conducted in the same way as the invasion assay, except for coating with Matrigel. Each type of cell was assayed in triplicate.

### Cell cycle and apoptosis analysis

For cell cycle analysis, cells were collected and fixed with ethanol (final concentration 70%) at −20°C for overnight. The fixed cells were reconstituted in Propidium Iodide (P4170, Sigma–Aldrich) staining solution (containing 20 ug/ml propidium iodide and 10ug/ml RNaseA) at 37°c for 30min. The stained cells were analyzed using flow cytometry using FL-2A to score the DNA content of the cells. Apoptosis assay was performed according to the manual of BD Annexin V-FITC apoptosis detection Kit (Cat: 556547, BD Biosciences, Franklin Lakes, NJ USA). Briefly, the harvested cells were washed twice with coldPBS and then resuspended in 1× Annexin binding Buffer. Then these cellswere stained with annexin V conjugated with FITC at room temperature inthe dark for 30min. And these stained cells were analyzed using flow cytometry as soon as possible (within 1 hour).

### Soft agar colony formation assays

A bottom layer of 0.5ml of 0.8% agar in DMEM medium containing 10% FBS was prepared and allowed to solidify and cellswere seeded in a top layer of 0.4% agar in DMEM medium containing 10% FBS at a density of 5×10^2^ cells per well in 24-well plate (duplicates were performed). For this TE9 cells (5×10^2^)were transfected with siNS or siZNF282 and suspended in culture medium containing 0.4% agar. Cells were incubated at 37°C (medium was added periodically) and colonies formed after 2 weeks were stained with crystal violet and then counted.

### Sphere formation assay

1 × 10^3^ cells were cultured in defined serum-free medium composed of DMEM + F12 medium, 20 ng/ml of EGF (epidermal growth factor; R&D Systems), 20 ng/ml of bFGF (basic fibroblast growth factor; R&D Systems), and B27supplement (R&D Systems). The cells were seeded in an Ultra-Low Attachment 96 well plate (Corning 3471). Spheroids were resuspended to form secondary and tertiary spheroids. The number of spheroids was counted after 14 days.

### Xenograft Experiments

TE9 cells (1×10^6^)infected with lentivirus expressing a non-specific (shNS) or ZNF282 shRNA (sh ZNF282) were suspended in 100 μl Matrigel/PBS (50:50 mixture) and injected subcutaneously into the right flank of 6-week-old female NOD-SCID mice (Orient Bio, Korea). Tumors were measured every week usinga digital caliper and the volumes were calculated according the formula: volume = length × width^2^/2. All animal experiments were conducted with the approval of the InstitutionalAnimal Care and Use Committee of Laboratory Animal Research Center at Samsung Biomedical Research Institute.

### Plasmids

cDNA encoding E2F1 from pRC/CMV-HA-E2F1 (gift from David M. Livingston, Harvard Medical School) was cloned into pSG5.HA and pGEX-4T-1. To generate the 5xE2F-TA-LUC reporter plasmid, oligonucleotides containing five E2F response elements (5′-CGC GTT TTG GCG CGT AAT TTG GCG CGT AAT TTG GCG CGT AAT TTG GCG CGT AAT TTG GCG CGT AAC-3′[sense] and 5′-TCG AGT TAC GCG CCA AAT TAC GCG CCA AAT TAC GCG CCAAAT TAC GCG CCA AAT TAC GCG CCA AAA-3′[antisense]) were cloned into pTA-LUC vector (Clontech). pSG5.HA- ZNF282 and pFLAG.CMV4- ZNF282 vectors were described previously [7].

### Protein interaction assays, immunoblot, and antibodies

For GST pull-down assays, HA epitope-tagged proteins were synthesized in vitro by using TNT-Quick coupled transcription/translation system (Promega) and incubated with immobilized GST-fusion proteins. After washing, bound proteins were analyzed by immunoblot with anti-HA antibody. For coimmunoprecipitation (CoIP) assays, 293T or TE10 cell extracts were immunoprecipitated by specific antibodies or control IgG and protein G Dynabeads (Invitrogen) as indicated in figure legends. The following antibodies were used in this study: anti- ZNF282 antibodies HPA024374 (Sigma) and D-13 (Santa Cruz Biotechnology); anti-E2F1 antibodies C-20 and KH95 (Santa Cruz Biotechnology); monoclonal anti-FLAG M2 antibody (Sigma); anti-tubulin antibody TU-02 (Santa Cruz Biotechnology); anti-HA antibody 3F10 (Roche).

### Chromatin Immunoprecipitation (ChIP) Assay

ChIP experiments were performed according to the procedure described previously [7, 29]. The cross-linked, sheared chromatin fractions were immunoprecipitated with anti-E2F1 antibody C-20 (Santa Cruz Biotechnology) or anti-ZNF282 antibody D-13 (Santa Cruz Biotechnology). The immunoprecipitated DNAs were amplified by qPCR using the following primers: CCNA1 promoter (nucleotides −4 to +164 relative to transcription start site), 5′-GCC AGT TGT TCC GGA CAC ATA-3′ (forward) and 5′-GAT CCA GGG TAC ATG ATT GCG-3′ (reverse); CDC6 promoter (nucleotides −148 to +30 relative to transcription start site), 5′-CTC TCT CAT TGG CTG TAA CTC-3′ (forward) and 5′-CAG CGG CAG CAG CAA ACT CCA-3′ (reverse).

### Statistical analysis

Statistical analyses were conducted using Pearson's χ^2^ tests, Fisher's exact tests, Cochan armitage trend test, ANOVA, Mann-Whitney tests, Tukey's HSD, and Duncan's test (as a *post hoc*test). Overall survival (OS) and disease free survival (DFS) were determined using the Kaplan- Meier method and the comparison was performed by using the log-rank or Breslow test as appropirate. Survivalwas measured from the date of surgery. The Cox proportional hazards model was used for multivariate analysis to evaluate the prognostic value of clinicopathologic factors. The hazard ratio (HR) and its 95% confidence interval (CI) were assessed for each factor. All tests were two sided, and *P*≤0.05 was considered statistically significant. All statistical analyses were performed usingSPSS software (SPSS Inc., Chicago, IL, USA).

## SUPPLEMENTARY MATERIAL FIGURES AND TABLE


