# Supplementary material for: ZNF282 (Zinc finger protein 282), a novel E2F1 co-activator, promotes esophageal squamous cell carcinoma
Source: Oncotarget. 2014 Oct 24;5(23):12260–72. doi: 10.18632/oncotarget.2630 (PMC4323012; doi:10.18632/oncotarget.2630)
Supplement: Supplementary file 1 [file oncotarget-05-12260-s001.pdf]

ZNF282 (Zinc finger protein 282), a novel E2F1 co-activator, promotes esophageal squamous cell carcinoma

Supplementary Material

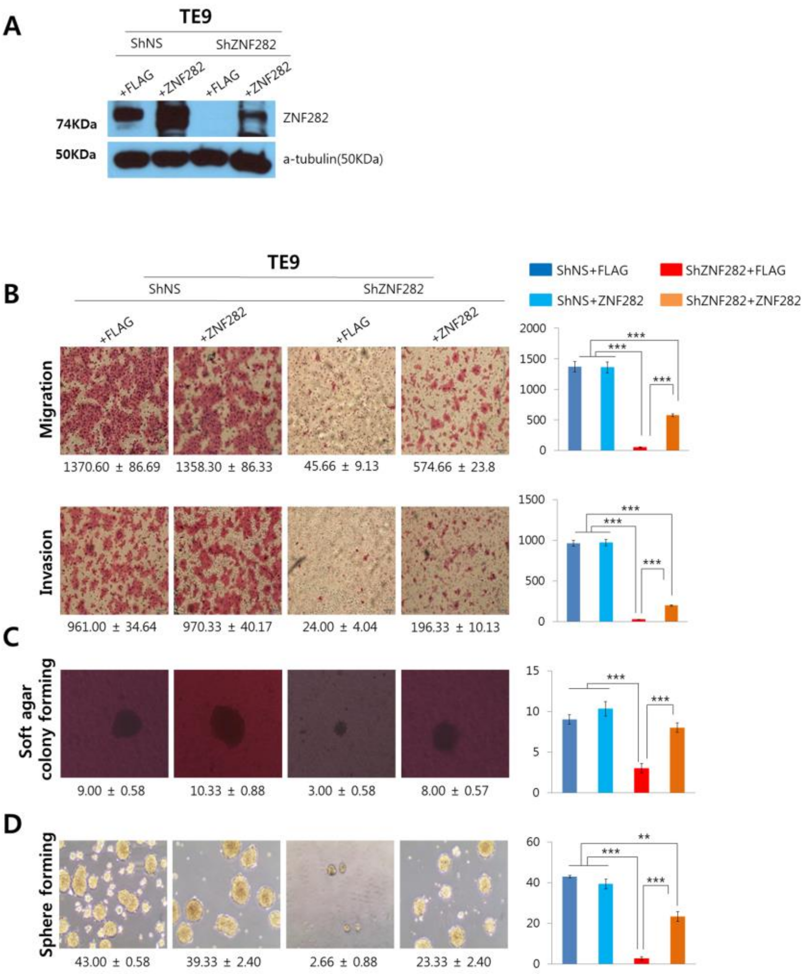

Supplementary Figure 1: (A) ZNF282 overexpression by transducing with ZNF282-FLAG was confirmed by Western blotting. (B-D) ZNF282 overexpression induced significant increase in migration, invasion (B), soft agar colony forming (C), and sphere forming (D) in TE9 cell lines that had been treated with shZNF282, while no significant change in shNS-treated TE9 cells. Number below the picture indicates mean value ± standard deviation. (*$p<0.05$, **$p<0.01$, ***$p<0.001$).

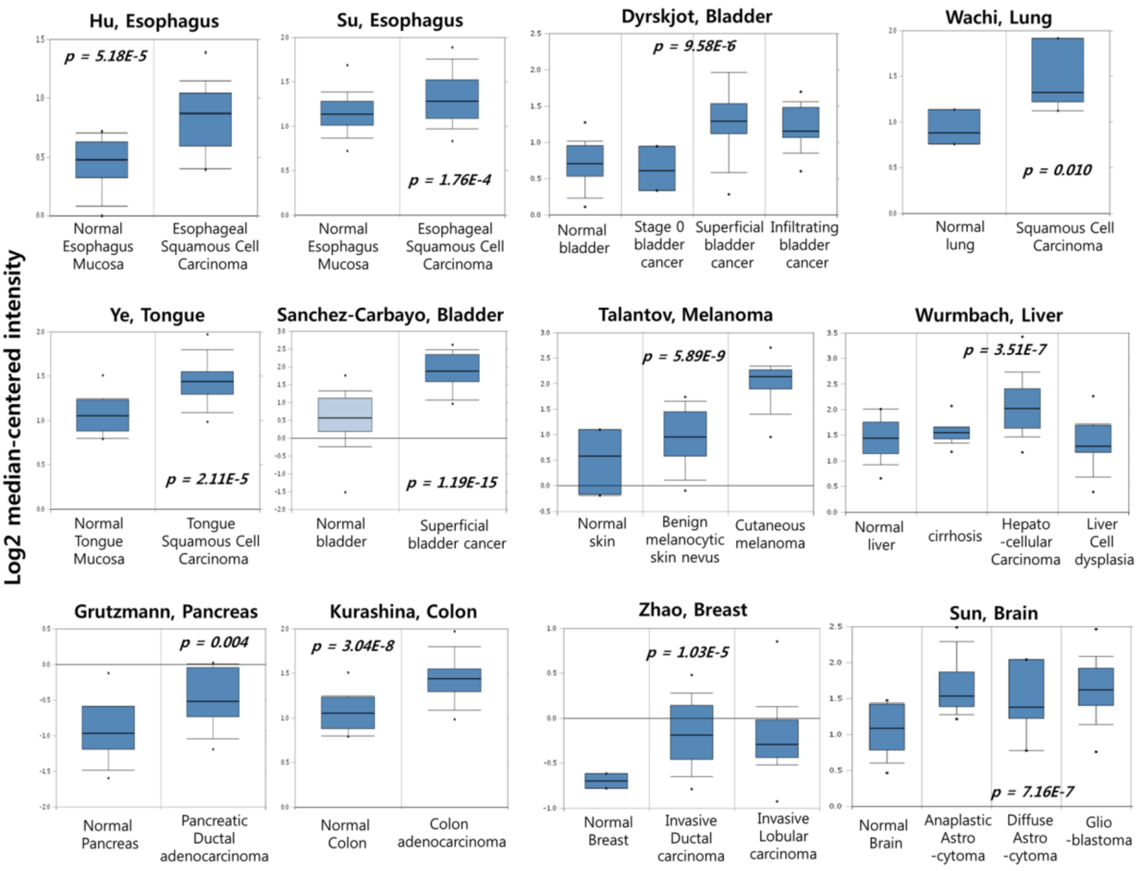

Supplementary Figure 2: Bioinformatics analysis of ZNF282 mRNA expression in human cancer samples using database at ONCOMINE (http://www.oncomine.org).

Table 1: Results of the immunohistochemical analysis of ZNF282 expression in normal and ESCC tissues

	Low expression	High expression	p value
Squamous cell carcinoma (n=165)	87 (52.7%)	78 (47.3%)	<0.001
Normal squamous epithelium (n=35)	33 (94.3%)	2 (5.7%)	
